# Laparoscopic versus Open Surgery in Complicated Appendicitis in Children Less Than 5 Years Old: A Six-Year Single-Centre Experience

**DOI:** 10.1155/2016/4120214

**Published:** 2016-09-25

**Authors:** R. Guanà, L. Lonati, S. Garofalo, N. Tommasoni, L. Ferrero, A. Cerrina, R. Lemini, C. Dallan, J. Schleef

**Affiliations:** Division of Pediatric General, Thoracic & Minimally Invasive Surgery, AOU Città della Salute e della Scienza, Regina Margherita Children's Hospital, Turin, Italy

## Abstract

*Introduction*. Acute appendicitis is the most common surgical emergency in the pediatric population. The peak incidence occurs in the first decade of life, while it is uncommon to face appendicitis in children younger than 5 years of age. Laparoscopy is now demonstrated to be the optimal approach also to treat complicated appendicitis, but in very young children this standardized operation is not always easy to perform.* Material and Methods*. From January 2009 to December 2015 we operated on 525 acute appendicitis, with 120 patients less than 5 years of age.* Results*. 90 children had a complicated appendicitis (localized or diffuse peritonitis): 43 (48%) were operated on by open approach and 47 (52%) by laparoscopy. The overall incidence of postoperative complications was greater in the open appendectomy group (63% versus 26%) and all severe complications requiring reintervention (6% of cases: 3 postoperative abscesses resolved with ultrasound guided percutaneous abscess drainage; 1 tubal surgery for salpingitis; 1 adhesion-related ileus requiring relaparotomy) were mostly associated with open surgery.* Conclusions*. Laparoscopic surgery resulted as the best approach for treating complicated appendicitis also in younger children, with minor and less severe postoperative complications compared to open surgery.

## 1. Introduction

Acute appendicitis in the pediatric population remains the most common surgical emergency [1]. The lifetime risk of developing an appendicitis is reported to be 6.7% in females and 8.7% in males [2].

The peak incidence occurs in the first and second decade of life, while it is uncommon to face appendicitis in children younger than 5 years of age [3].

The clinical presentation may be varied and often is similar to other medical conditions, so a misdiagnosis can be frequent and the most common one is usually gastroenteritis.

The examination of the abdomen can show different signs such as focal or diffuse tenderness, guarding, rebound, and mass, but these are better identified in older children.

Young children have instead specific anatomic and pathophysiologic elements for developing more often complicated appendicitis; moreover it has been established that children less than 6 years old usually are diagnosed with two or three days in delay [4].

In children and adults acute appendicitis can be treated with different surgical approaches.

The most used ones are the open appendectomy (OA), the laparoscopic appendectomy (LA), and the transumbilical laparoscopic-assisted appendectomy (TULAA).

Moreover new less invasive techniques, such as the natural orifice transluminal endoscopic surgery (NOTES), have been developed mostly for adults patients.

In recent years, many studies have been performed on this subject and have showed that the use of laparoscopy in acute appendicitis has increased up to 50–60%.

Comparing the operative time, the length of hospital stay, the return to normal activity, and the complications rate, laparoscopic techniques usually resulted as the best choice for the patient [5–8].

Laparoscopic appendectomy showed to be better than open appendectomy in complicated appendicitis as well [9, 10].

But in the specific age group of younger children, there are few reports that compare the two different approaches and the incidence of postoperative complications.

## 2. Patients and Methods

The medical records of 525 consecutive pediatric patients (less than 14 years old), which underwent appendectomy for acute appendicitis from January 2009 to December 2015 at our institution, were retrospectively reviewed.

Demographic data, histopathology reports, surgical procedure descriptions, and possible complications were extracted from clinical records and healthcare information system (Trakcare, InterSystem Corporation, Cambridge, MA, USA) (Table 1).

Our diagnostic and therapeutic protocol from about ten years was the following: if the patient was thought to have an acute appendicitis preoperatively diagnosed by physical, laboratory findings and ultrasound examination, antibiotic treatment was started immediately with third generation cephalosporins.

Antibiotics were continued 1 day postoperatively if the appendix was not perforated (2 days postoperatively if intra-abdominal serous fluid was signalled as intraoperative findings) or 7 days (associating gentamicin sulphate and metronidazole) if the appendix was necrotic or gangrenous or a localized or diffuse peritonitis was present.

Antimicrobial agents were eventually changed on the basis of the antibiogram report.

The appendectomies were performed with an open approach through a right lower quadrant incision or by laparoscopy, performed through a 3-trocar classical technique, depending on the skills of the surgeons with laparoscopic procedures.

Open appendectomy was performed through a right lower quadrant muscle-splitting incision; the appendix was tied at the base and then divided; the stump was not inverted.

Laparoscopic appendectomy was accomplished using an open technique and a standard 3-trocar technique: the appendix was divided at the base using an endoscopic stapling device or tied at the base with endoloops and divided; it was removed from the abdomen in a plastic bag through the umbilical port.

The use of right lower quadrant drain was extremely limited; in fact tube insertion was not routinely done, but only in extremely extensive abscess or in the pelvic or retrocolic regions at risk for recurrence of abscessualization.

Statistical analysis to compare results was performed using SPSS 15 (SPSS Inc., Chicago, IL, USA).

Continuous variables have been compared by Student's *t*-test after assessment of normal parametric distribution by Shapiro-Wilk test.

Distribution of noncontinuous variables in the groups has been compared by 2 × 2 Fisher exact test (Table 2).

## 3. Results

From January 2009 to December 2015 we operated on 525 acute appendicitis, with patient median age of 9 years old (64% male, 36% female).

357 (68%) were nonperforated appendicitis (M 65%, F 35%) while 168 (32%) had a perforated appendicitis (M 55%, F 45%).

Laparoscopic technique was adopted in 253 patients (170 with 67% nonperforated appendicitis, 83 with 33% perforated appendicitis), while open approach was adopted in 272 patients (187 with 69% nonperforated appendicitis, 94 with 31% perforated appendicitis), depending on the surgeon's experience carrying out safely one approach rather than the other.

Twenty patients (8%) among 253 were converted to open surgery due to the difficulty to understand the exact anatomy of the appendix and its relationship with the ileocecal region. Three patients were in the age group lower than 5 years old, all with complicated appendicitis.

120 patients were less than 5 years old (30 under 3 years, 3 less than 2 years of age) and 90 children had a complicated appendicitis (with perforated appendicitis and localized or diffuse peritonitis as intraoperative findings ): 43 (48%) were operated on by open approach and 47 (52%) by laparoscopy.

Median length of hospital stay was 8.5 days (range 6–17 days).

Total rate of postoperative complications was 43%, with 63% of postoperative complications for OA and 26% for LA.

**Figure 1:**
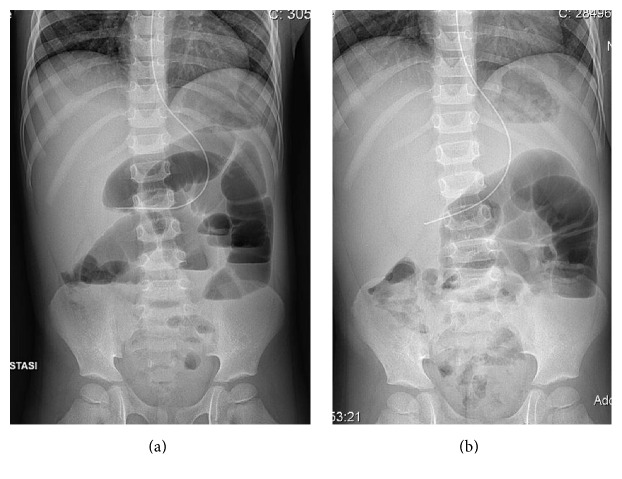
SBO complicating OA, needing relaparotomy.

**Figure 2:**
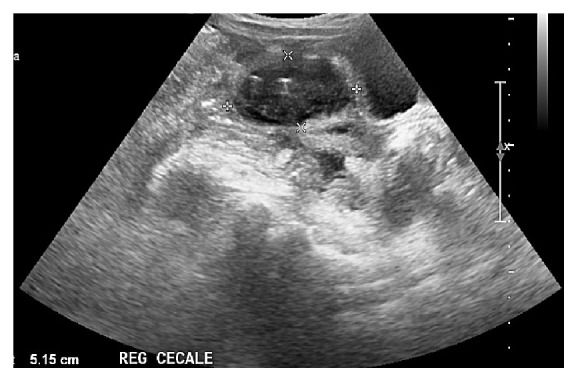
Residual pelvic abscess ultrasound image in OA group.

In the OA group, we reported persistent postoperative fever (more than 4 days, needing changing of antimicrobial agent) in 10 cases; wound infection with partial wound dehiscence in 3 patients; postoperative intra-abdominal abscess (pelvic, perirenal, right iliac fossa abscess and tubaric abscess) in 6 cases (3 necessitated percutaneous drainage, while the tubaric one requested laparoscopic right tubal removal); small bowel occlusion in 7 patients, with one relaparotomy for obstinate abdominal adhesion (Figures 1 and 2).

We registered one enterocutaneous fistula resolved conservatively with total parenteral nutrition prolonged for two weeks.

In the LA group, 8 patients suffered from persistent postoperative fever; 3 had postoperative intra-abdominal abscess (pelvic and retrocolic) resolved with antibiotics and 1 underwent small bowel occlusion resolved with nasogastric tube insertion.

The overall incidence of complications was greater in the open appendectomy group (63% versus 26%; *p* = 0.002) and all severe complications requiring reintervention (6% of cases: 3 postoperative abscesses resolved with ultrasound guided percutaneous abscess drainage; 1 tubal surgery for salpingitis; 1 adhesion-related ileus requiring relaparotomy) were mostly associated with open surgery.

## 4. Discussion

Clinical presentation of pediatric appendicitis is rather typical: the pain is always present, initially located at the periumbilical area and afterwards at the right iliac fossa; in most of the cases vomit and lack of appetite follow the pain, associated to fever, which is also a very common find.

The examination of the abdomen, instead, can show different signs such as focal or diffuse tenderness, guarding, rebound, and mass, but these are better identified in older children.

Given this presentation, a misdiagnosis can be frequent and the most common one is the gastroenteritis.

Sometimes the actual diagnosis is delayed, and thus the severity of the condition increases, leading to a complicated appendicitis.

The laboratory evaluation of white blood cell count and C-reactive protein (CRP) and the use of radiological techniques such as ultrasonography or computed tomography can help in diagnosing correctly and timely [11, 12].

However, younger children have specific anatomic and pathophysiologic elements for developing complicated appendicitis, a rather short appendix with a thin appendiceal wall and an undeveloped omentum, and toddlers are usually particularly exposed to gastrointestinal viruses that leave a transient immunosuppression with subsequent possible bacterial overgrowth.

Children less than 6 years old usually are diagnosed with two or three days in delay.

Close observation and evaluation are mandatory for not delaying time before surgery.

Laparoscopy is now demonstrated to be the optimal approach also to treat complicated appendicitis, but in very young children this standardized operation is not always easy to perform.

Pneumoperitoneum in infants should be of low pressure for possible haemodynamic effects and so the working space could be very limited.

Also the use of endoscopic mechanical staplers could be limited by the abdominal cavity dimensions.

There have been many prospective randomized controlled trials in the adult population comparing LA with OA in complicated appendicitis, while in the pediatric population data are lacking.

Our data support the safety and feasibility of LA also in children less than 5 years old in particular regarding the low rate of complications of LA compared to OA.

Given the retrospective nature of the study, selection of cases submitted to LA or OA may be biased by factors as age at presentation or symptoms.

On the contrary, no significant differences have been found in the presence of peritonitis, kind of histology, or in complications between the two groups.

Both time to return to normal activities and regular diet appear reduced by 25% using the LA approach.

Operative time is only moderately increased in the LA approach (17%).

The number of cases with postoperative abscesses is more than halved, though not significantly (this can be due to the overall low incidence of abscess in both groups).

Those complicated by postoperative ileus are significantly lower.

## 5. Conclusions

Early childhood is particularly affected by complicated appendicitis because of the nonspecificity of signs and symptoms and the rapid evolution of bacterial overgrowth.

Laparoscopic surgery resulted as the best approach for treating complicated appendicitis also in younger children, with minor and less severe postoperative complications compared to open surgery.

## Supplementary Material

Summarizing table of all the data collected on the 120 patients less than 5-years-old (in red font the children less than 2-years-old).

## Figures and Tables

**Table 1:** Descriptive table of patients demographic data and clinical presentation.

	Variable	Numbers	Percentage
Mean age (years)		4.16 y (from 2 to 5)	
Gender	Male	83	69.2%
	Female	37	30.8%
Weight (Kg)		15.97 Kg (from 12 to 19)	
Preop presentation	Pain, fever	13	10.8%
	Pain, fever, diarrhea	6	5.0%
	Pain, fever, vomiting	10	8.3%
	Pain, fever, diarrhea, vomiting	1	0.8%
	Acute abdomen	90	75%
Preop peritonitis	Yes	69	57.5%
	No	51	42.5%
Surgical approach	Open appendectomy	55	45.8%
	Laparoscopic appendectomy	65	54.2%
Complicated appendicitis	Yes	90	75%
	No	30	25%
Postop abscess	Yes	9	7.5%
	No	111	92.5%
Postop ileus	Yes	8	6.7%
	No	112	93.3%

**Table 2:** Percentage and statistical analysis results (^*∗*^Student's *t*-test).

	OA	LA	*p*
Number of patients	55	65	
Age	3.8 ± 1.0	4.4 ± 0.6	<0.001^*∗*^
Preop peritonitis	35 (63.6%)	34 (52.3%)	0.267
Complicated/uncomplicated	43 (78.1%)	47 (72.3%)	0.529
Operative time (min)	59.0 ± 11.0	69.0 ± 13.8	<0.001^*∗*^
Return to normal activities time (days)	7.2 ± 2.0	5.4 ± 1.7	<0.001^*∗*^
Return to regular diet time (days)	6.8 ± 2.2	5.2 ± 1.6	<0.001^*∗*^
Pain control (IV)	Acetaminophen, 12	Acetaminophen, 18	Needing more than 1 pain drug <0.001 Needing tramadol 0.529
	Tramadol, 0	Tramadol, 40	
	Both, 43	Both, 7	
Postop abscess	6 (10.9%)	3 (4.6%)	0.298
Postop ileus	7 (12.7%)	1 (1.5%)	0.023
Histology	Empyematous, 5	Empyematous, 6	0.683
	Phlegmonous, 7	Phlegmonous, 12	
	Gangrenous, 43	Gangrenous, 47	
